# Iron status in women with infertility and controls: a case-control study

**DOI:** 10.3389/fendo.2023.1173100

**Published:** 2023-06-08

**Authors:** Iris Holzer, Johannes Ott, Klara Beitl, Daniel Mayrhofer, Florian Heinzl, Johanna Ebenbauer, John Preston Parry

**Affiliations:** ^1^ Clinical Division of Gynecologic Endocrinology and Reproductive Medicine, Medical University of Vienna, Vienna, Austria; ^2^ Department of Obstetrics and Gynecology, Medical University of Vienna, Vienna, Austria; ^3^ Department of Obstetrics and Gynecology, Louisiana State University Health-Shreveport, Shreveport, LA, United States; ^4^ Parryscope and Positive Steps Fertility, Madison, MS, United States

**Keywords:** unexplained infertility, sterility, iron deficiency, serum ferritin, thyroid, iron deficiency, thyroid antibodies

## Abstract

**Background:**

Iron deficiency is a common problem in subfertile women. The influence of iron status on unexplained infertility is unknown.

**Methods:**

In a case-control study, 36 women with unexplained infertility and 36 healthy non-infertile controls were included. Parameters of iron status including serum ferritin and a serum ferritin <30 µg/dL served as main outcome parameters.

**Results:**

Women with unexplained infertility demonstrated a lower transferrin saturation (median 17.3%, IQR 12.7-25.2 versus 23.9%, IQR 15.4-31.6; *p=* 0.034) and a lower mean corpuscular hemoglobin concentration (median 33.6 g/dL, IQR 33.0-34.1 versus 34.1 g/dL, IQR 33.2-34.7; *p=* 0.012). Despite the fact that there was no statistically significant difference in median ferritin levels (*p=* 0.570), women with unexplained infertility had ferritin levels <30µg/L more often (33.3%) than controls (11.1%; *p=* 0.023). In a multivariate model, unexplained infertility and abnormal thyroid antibodies were associated with ferritin <30µg/L (OR 4.906, 95%CI: 1.181-20.388; *p=* 0.029 and OR 13.099; 2.382-72.044; *p=* 0.029, respectively).

**Conclusion:**

Ferritin levels <30µg/L were associated with unexplained infertility and might be screened in the future. Further studies with a focus on iron deficiency and iron treatment on women with unexplained infertility are warranted.

## Introduction

The overlap between nutritional status and unexplained infertility remains controversial and routinely is a matter for debate. One of the most important deficiencies commonly identified in both subfertile women as well as those not trying to conceive is iron deficiency. Sufficient iron supply is an important proxy for wellness, regulating numerous physiological and cellular processes (1). Given the widespread effects of iron deficiency on so many systems, there is concern that inadequate levels could contribute to infertility or recurrent miscarriages.

Iron homeostasis is frequently assessed through serum ferritin levels (2). The World Health Organization defines iron deficiency as ferritin <15 µg/L, but it has been argued that this cut-off should be increased to <30µg/L to improve the sensitivity of this parameter in populations with and without diseases (3). In a review about iron status in European females of childbearing age, median or geometric mean serum ferritin levels were assessed at 26-38 µg/L and about 40-55% of this females had low iron stores considered as serum ferritin levels ≤ 30 µg/L (3).It is well known that females suffer iron deficiency more frequent than males, which is mainly caused by the blood loss during menstruation. With the average blood loss of a normal menstruation about 16 mg iron are lost. It is reasonable that females with abnormal uterine bleeding (menorrhagia) were found to have higher chances for iron deficiency and iron deficiency resulting in anemia (4–6).

Concerning female fertility, Clancy et al. demonstrated that women with higher values of hemoglobin have more often an endometrium of more thickness (7). The authors concluded that improved health, as reflected by iron status, allowed females to get a endometrium of more thickness despite the fact that endometrium of more thickness was related with heavier menstruation and more blood loss (7). Among other results of a large study investigating the association between iron supplementation and hair loss, the cases of seven females, who became pregnant during supplemental iron intake, were investigated (8). Despite the small number of females, the authors of this publication propose an intriguing association between iron status and the ability to get pregnant (2, 8). Furthermore, a large prospective study over an 8-year period, which included 18.555 premenopausal females who regularly take iron supplements, demonstrated a significantly lower chance of infertility than in females without consumption of iron supplements (9). Additionally, in a recently published study on 84 females who suffered from recurrent pregnancy loss and 153 healthy controls, females with recurrent pregnancy loss demonstrated lower serum ferritin levels than the control group (39.9µg/L vs. 62.2µg/L) and had a more frequent occurrence of low iron status which was defined as ferritin levels <30 µg/L (35.7% vs. 13.7%) (10).

The role of serum auto-antibodies to infertility is still under debate, but there are studies about an association with iron deficiency, which is likely due to the associated comorbidities of autoimmune gastritis and coeliac disease (11). Thus, thyroid autoimmunity, which also has a well-known effect on thyroid physiology, might have an additional impact on infertility, even in euthyroid women (12–14).

There are no epidemiologic data on iron status and females with infertility/subfertility. Thus, the aim of our study was to evaluate the iron status in females with unexplained infertility in detail and to compare the findings to a control group of healthy females without infertility. In addition, we also focused on serum parameters for autoimmune thyroiditis.

## Materials and methods

### Patient population

This case-control study was performed at the Clinical Division of Gynecologic Endocrinology and Reproductive Medicine of the Medical University of Vienna, Austria. From November 2021 to September 2022, females with unexplained infertility and a control group of healthy females were included. All women were aged 18-40 years and had menstrual cycles of 24 to 38 days without signs of abnormal uterine bleeding in the last twelve cycles before enrollment in the study, which is considered normal (11). Use of any iron supplementation within the last six months was an exclusion criterion for all participants. The definition of unexplained infertility was determined as the non-occurrence of pregnancy after 12 months of regular unprotected sexual intercourse without presence of an identifiable cause (15). Potential causes for infertility constituting grounds for exclusion included oligoovulation, anovulation, blocked fallopian tubes (bilateral occlusion with hysterosalpingography or hysterosalpingo-contrast sonography), dysmenorrhea (with numeric rating scale >3), known endometriosis, fibroids, intracavitary abnormalities, or impairment of semen parameters (16). Participants of the healthy control group who volunteered did not suffer from primary or secondary infertility and were also otherwise healthy.

The study was approved by the Ethics Committee of the Medical University of Vienna (IRB number 1400/2021) and was conducted in accordance with the Declaration of Helsinki and the guidelines of Good Clinical Practice. Written informed consent was obtained for all cases. All records were anonymized and de-identified prior to the analyses.

### Outcome parameters

The main outcome parameter was serum ferritin (presented as a numerical and a categorical parameter). A serum ferritin level <30 µg/L was considered as status of iron deficiency (3). Other serologic parameters included additional markers of iron status (transferrin, transferrin saturation, C-reactive protein, CRP), general hematological parameters (erythrocyte count, hemoglobin, hematocrit, mean corpuscular volume, mean corpuscular hemoglobin, mean corpuscular hemoglobin concentration, thrombocyte count, and leucocyte count), thyroid peroxidase antibodies (TPO-Ab), thyroglobulin antibodies (TG-Ab), follicle-stimulating hormone (FSH), luteinizing hormone (LH), anti-Mullerian hormone (AMH), total testosterone, androstenedione, dehydroepiandrosterone-sulphate (DHEA-S), thyroid-stimulating hormone (TSH), sexual hormone binding globulin (SHBG) and 25-hydroxyvitamin D3 (25OHD3). On the second to the fifth day of the menstruation cycle all included females had to undergo baseline blood sampling. The determination of all relevant serum parameters was performed at the Department of Laboratory Medicine, Medical University of Vienna, according to ISO 15189 quality standards. Finally, the following general patient characteristics were evaluated: age, body mass index (BMI), smoking status, duration of infertility in months, and primary versus secondary infertility.

### Sample size calculation

Anticipating a mean difference in ferritin levels of 10 ng/mL with a standard deviation of ±10 ng/mL between tested groups and using an alpha of 0.01 with a power of 95%, a sample size of 36 per group was needed.

### Statistical analysis

Data are present as median and interquartile range (IQR) for numerical parameters and as numbers (frequencies) for categorical data. Differences between groups were tested using unpaired t-tests/Fisher’s exact tests and Kruskal Wallis tests. Moreover, a binary logistic regression model was used to evaluate parameters associated with serum ferritin <30 µg/L. Univariate analyses were calculated and all significant parameters were entered into a multivariate model. For these analyses, odds ratios (OR) and 95% confidence intervals (95% CI) are provided. Statistical analyses were conducted with SPSS 26.0 and/or the open-source software “R”. P-values <0.05 were defined statistically significant.

## Results

General characteristic data of the study group suffering unexplained infertility (*n=* 36) and the healthy control group (*n=* 36) are listed in **Table 1**. Most parameters did not differ between the two study cohorts. However, in the study group, a higher median age (33.0 years, IQR 29.9-36.2 versus 28.4 years, IQR 27.1-31.1; *p<* 0.001), higher TSH levels (1.85 µIU/mL, IQR 1.13-2.19 versus 1.33 µIU/mL, IQR 1.10-1.79; p= 0.039) and higher prolactin levels (17.3 ng/mL, IQR 10.9-21.1 versus 12.5 ng/mL, IQR 8.7-17.2; p= 0.008) were found.

**Table 1:** Basic subject characteristics and results of hormonal testing.

	Unexplained infertility	Healthy volunteers (controls)	P
N	36	36	–
Age (years) *	33.0 (29.9;36.2)	28.4 (27.1;31.1)	<0.001
BMI (kg/m^2^) *	24.0 (20.7;27.4)	21.2 (20.1;22.3)	0.012
Primary infertility ^#^	18 (50.0)	–	–
Duration of infertility (months) *	24.0 (14.7;48.0)	–	–
Smoking ^#^	8 (22.2)	4 (11.1)	0.860
TPO-Ab (IU/mL) *	0 (0,9)	4.5 (0;12.8)	0.108
TG-Ab (IU/mL) *	13 (13;22)	14(13;16)	0.090
Abnormal TPO-Ab and/or TG-Ab ^#^	5 (13.9)	4(11.1)	1.000
TSH (µIU/mL) *	1.85 (1.13;2.19)	1.33 (1.10;1.79)	0.039
LH (mIU/mL) *	6.8 (5.0;9.1)	6.5 (4.3;8.3)	0.457
FSH (mIU/mL) *	6.6 (5.4;8.8)	6.3 (5.4; 7.6)	0.341
Prolactin (ng/mL) *	17.3 (10.9;21.1)	12.5 (8.7;17.2)	0.008
Testosterone (ng/mL) *	0.27 (0.18;0.36)	0.22 (0.12;0.30)	0.124
Androstenedione (ng/mL) *	1.15 (0.83;1.58)	1.02 (0.88;1.29)	0.341
DHEA-S (µg/mL) *	2.17 (1.57;2.73)	2.28 (2.01;3.14)	0.164
SHBG (nmol/L) *	53.8 (40.7; 76.0)	70.9 (56.2;97.2)	0.009
AMH (ng/mL) *	2.92 (1.39;4.21)	3.07 (2.27;4.39)	0.287
25-OH vitamin D (nmol/L) *	67.3 (42.7;79.6)	79.2 (63.);88.8)	0.018

Data are provided as * median (IQR) for numerical parameters and ^#^ n (%) for categorical parameters. "- ", not applicable.

**Figure 1:**
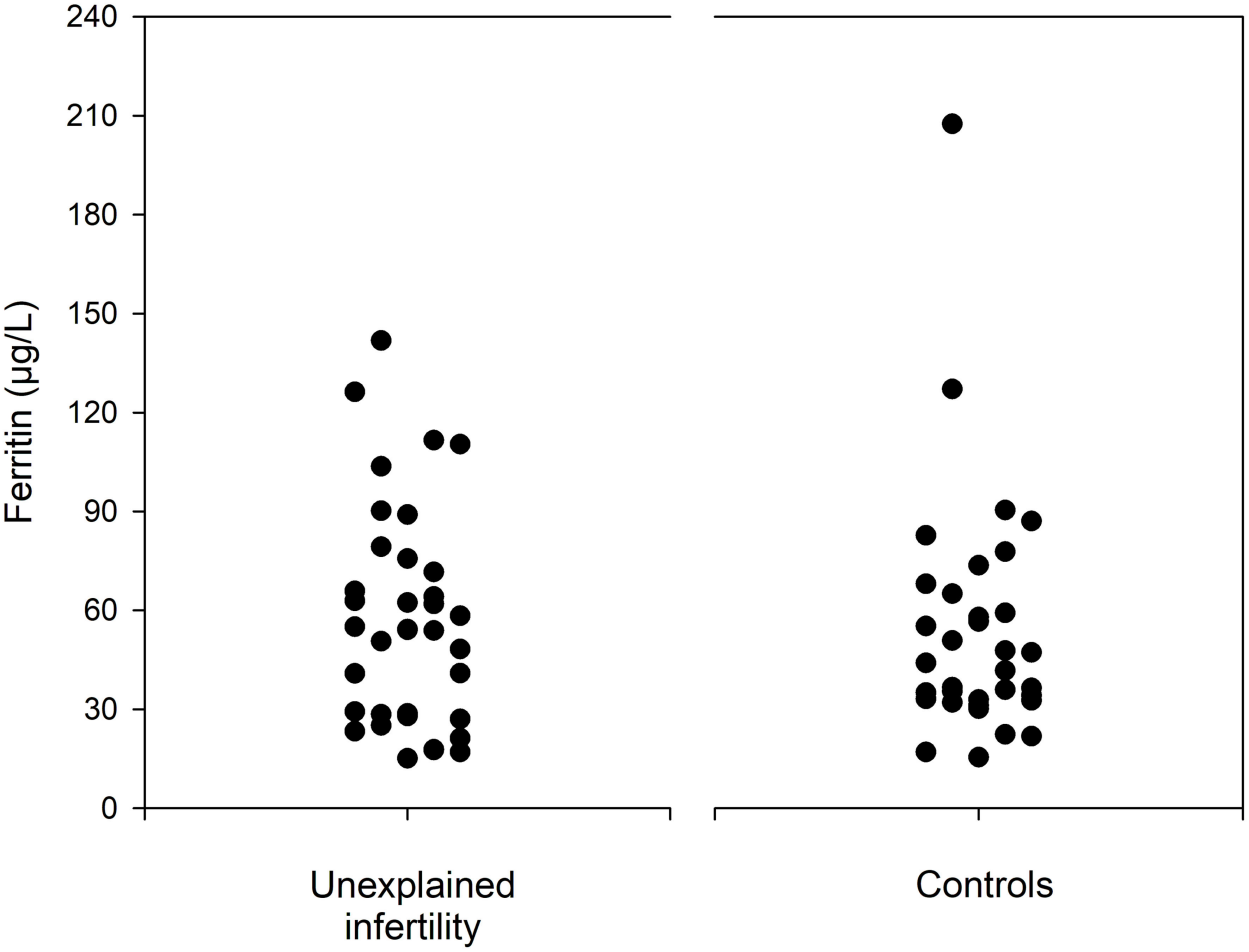
Serum ferritin levels in Women with unexplained infertility and controls.

**Table 2** shows details of serological outcome parameters. Notably, the median serum iron was higher in the control group than in the study group (62.5 µg/dL IQR 52.3-85.0 versus 91.0 µg/dL, IQR 65.3-104.0; *p=* 0.003). In contrast, women with unexplained infertility demonstrated a lower transferrin saturation (median 17.3%, IQR 12.7-25.2 versus 23.9%, IQR 15.4-31.6; *p=* 0.034), a lower mean corpuscular hemoglobin concentration (median 33.6 g/dL, IQR 33.0-34.1 versus 34.1 g/dL, IQR 33.2-34.7; *p=* 0.012). A higher median CRP level was found in women with unexplained infertility (median 1.25 mg/L, IQR, 0.53-2.18) versus 0.40 mg/L, IQR 0.00-1.00; *p<* 0.001). Despite the fact that there was no statistically significant difference in median ferritin levels (*p=* 0.570), women with unexplained infertility had ferritin levels <30µg/L more often (33.3%) than controls (11.1%; *p=* 0.023). **Figure 1** provides data on serum ferritin levels in women with unexplained intertility and controls.

**Table 2:** Iron status.

	Unexplained infertility	Healthy volunteers (controls)	P
Erythrocyte count (T/L) *	4.6 (4.3;4.8)	4.3 (4.2;4.5)	0.007
Hemoglobin (g/dL) *	13.6 (12.4;14.2)	13.1 (12.6;13.6)	0.207
Hematocrit (%) *	40.1 (38.7;41.5)	38.5 (37.7;40.2)	0.056
Mean corpuscular volume (fl) *	88.5 (85.0;90.8)	88.7 (86.4;91.0)	0.585
Mean corpuscular hemoglobin (pg) *	33.6 (33.0;34.1)	34.1 (29.1;31.2)	0.180
Mean corpuscular hemoglobin concentration (g/dL) *	33.6 (33.0;34.1)	34.1 (33.2;34.7)	0.012
Thrombocyte count (G/L) *	267.0 (224.8;285.0)	242.5 (218.0;287.0)	0.347
Leucocyte count (G/L) *	6.3 (4.9;7.5)	6.0 (5.0;6.8)	0.673
Iron (µg/dL) *	62.5 (52.3;85.0)	91.0 (65.3;104.0)	0.003
Transferrin (mg/dL) *	273.5 (253.0;300.5)	266.0 (241.3;305.3)	0.517
Transferrin saturation (%) *	17.3 (12.7;25.2)	23.9 (15.4;31.6)	0.034
Ferritin (µg/L) *	54.1 (28.0;76.6)	42.9 (32.5;63.6)	0.570
Ferritin <30µg/L ^#^	12 (33.3)	4 (11.1)	0.023
C-reactive protein (mg/L) *	1.25 (0.53;2.18)	0.40 (0.00;1.00)	<0.001

Data are provided as * median (IQR) for numerical parameters and ^#^ n (%) for categorical parameters.

In the whole study population, serum ferritin levels <30µg/L were found in 16 women (22.2%). In order to assess associated parameters, a multivariate binary logistic regression model was performed referring to univariate analyses (**Table 3**). In the final multivariate model, abnormal TPO-antibodies and/or TG antibodies (OR 13.099, 95%CI 2.382-72.044; *p=* 0.003) and the presence unexplained infertility (OR 4.906, 95% CI 1.181-20.388; *p=* 0.029) were independently associated with an increased chance for ferritin levels <30µg/L.

**Table 3:** Factors associated with ferritin <30µg/L. Results of a univariate analyses followed by a multivariate binary logistic regression model.

			Univariate analysis		Multivariate analysis	
	Ferritin <30µg/L (n= 16)	Ferritin ≥30µg/L (n= 56)	OR (95%CI)	p	OR (95%CI)	P
Age (years)*	33.4 (28.0;36.7)	30.6 (27.6;32.7)	1.143 (0.985;1.326)	0.078	–	–
BMI (kg/m^2^)*	23.2 (20.4;27.4)	21.9 (20.4;24.8)	1.092 (0.953;1.251)	0.207	–	–
TSH (µIU/mL)	1.7 (1.3;2.0)	1.4 (1.1;2.1)	1.515 (0.860;2.670)	0.150	–	–
Abnormal TPO-Ab and/or TG-Ab ^#^	6 (37.5)	3 (5.4)	10.600 (2.268;49.537)	0.003	13.099 (2.382;72.044)	0.003
Prolactin (ng/mL)	17.8 (12.6;22.1)	13.8 (9.4;18.1)	1.091 (0.996;1.195)	0.062	–	–
SHBG (nmol/L)	53.9 (40.9;97.2)	64.9 (45.5;81.1)	0.994 (0.979;1.009)	0.426	–	–
25-OH vitamin D (nmol/L)	67.3 (48.5;79.4)	75.0 (61.4;88.6)	0.984 (0.960;1.008)	0.198	–	–
Unexplained infertility	12 (75.0)	24 (42.9)	4.000 (1.147;13.951)	0.030	4.906 (1.181;20.388)	0.029
Constant	–	–	–	–	0.070	<0.001

"- ", not applicable.

Details of hematologic parameters in females with serum ferritin levels ≥30µg/L and <30µg/L are provided in the **Supplementary Table 1**. In the latter group, significantly lower median levels of serum hemoglobin levels, corpuscular hemoglobin concentration, mean corpuscular volume, mean corpuscular hemoglobin concentration, whereas serum transferrin was higher (*p<* 0.05).

## Discussion

We provided a detailed evaluation of iron status in females with unexplained infertility in comparison to a healthy control group. We assume that this study is the first to demonstrate an association of female iron deficiency determined as serum ferritin levels <30µg/L with unexplained infertility. This stands in accordance with a study on iron consumption and fertility among North American and Danish pregnancy planners that showed that the iron consumption in sum did not show an association with female infertility, but that there were some hints for a positive association among females having high chances for iron deficiency (17). Of interest, in an animal model with 15 rats with iron-deficiency after low-iron food and a control cohort of 15 rats with normal iron consumption, there was a significantly lower pregnancy rate in the group with the iron-deficiency rats, suggesting an important impact from iron deficiency on unexplained infertility (18). In addition, in a large cohort study, it was shown that females who consumed iron supplements had a significantly lower chance of ovulatory infertility than females who did not consume iron supplements (relative risk 0.60, 95%CI 0.39-0.92) (8). This potential etiology was inherently excluded through definition for our population of women with unexplained infertility, as ovulatory dysfunction had been ruled out. However, all of these results suggest that the effect of female iron status impact on reproductive function starts before pregnancy, over which there is a well know need for a substantial increase in iron consumption (19).

Causes for the link between iron deficiency and sterility remain hypothetical. Though the absolute amounts are small, it should be noted that the developing follicle also has increasing iron demands. Transferrin is expressed and synthesized by granulosa cells themselves and thereby iron metabolism affects follicle maturation and ovulation (9, 20).

In addition, our study demonstrated that increased serological markers for thyroid autoimmunity were also independent predictors for serum ferritin levels <30 µg/L. It is known that patients with autoimmune thyroiditis are often iron-deficient, which is due to the fact that the associated comorbidities autoimmune gastritis and coeliac disease result in decreased iron absorption and iron deprivation. In two-third of females with existent symptoms of hypothyroidism despite adequate levothyroxine treatment, reaching a serum ferritin above 100 µg/L improved symptoms (11, 21).

It is also well known that chronic inflammation has a major impact on iron homeostasis (22). The confounding influences of the acute -phase response make it challenging to interpret most iron indicators in case of acute infection and inflammation (23). Chronic inflammation might be the cause for the increased median serum iron level in the study group (62.5 µg/dL IQR 52.3-85.0 versus 91.0 µg/dL, IQR 65.3-104.0; *p=* 0.003) as well as the higher median CRP level in the study group (median 1.25 mg/L, IQR, 0.53-2.18) versus 0.40 mg/L, IQR 0.00-1.00; *p<* 0.001), despite the higher frequency of ferritin levels <30µg/L. Notably, increased ferritin levels during infection and inflammation may also represent an important host defense mechanism. Ferritin plays a major role in immune dysregulation, specifically in case of massive hyperferritinemia, acting directly in an immune suppressive and pro-inflammatory way (24). In contrast, in our study group, women with unexplained infertility revealed a lower transferrin saturation (median 17.3%, IQR 12.7-25.2 versus 23.9%, IQR 15.4-31.6; *p=* 0.034) and a lower mean corpuscular hemoglobin concentration (median 33.6 g/dL, IQR 33.0-34.1 versus 34.1 g/dL, IQR 33.2-34.7; *p=* 0.012). In our study cohort of females with serum ferritin <30µg/L, typical alterations of hematologic iron-related parameters were present including lower hemoglobin and mean corpuscular volume levels. However, transferrin saturation did not differ between the groups in a statistical significant manner (**Supplementary Table 1**). Anemia of inflammation most often presents as normocytic, normochromic anemia linked to decreased transferrin saturation (<20%) but increased serum ferritin levels (25, 26). It might be assumed that a lower transferrin saturation and a lower mean corpuscular hemoglobin concentration in our study group suffering unexplained infertility can be due to a mild chronic inflammation even in absence of anemia.

Despite knowledge of the link between an abnormal iron status and chronic inflammation, data about chronic inflammation and unexplained infertility are scarce. Chronic inflammation, that is usually diagnosed by serum high-sensitivity CRP levels has been linked to unfavorable reproductive results, as reduced fertility, recurrent miscarriage, and recurrent implantation failures after *in-vitro*-fertilization (IVF) (27, 28). There might be an association of several factors linked to higher serum levels of CRP and infertility, particularly severe overweight, endometriosis or PCOS (29–31). In regard to our study group, only patients with unexplained infertility were included. The higher CRP levels of our study group could therefore not be explained by these factors associated with infertility. The results of a recently published study on 781 couples with unexplained infertility suggest that a chronic inflammatory status could raise the chance of pregnancy loss but may not affect the clinical pregnancy rate in females with unexplained infertility that are under treatment for ovulation induction followed by intrauterine insemination because of unexplained infertility (32). Although moderate inflammation is present in 20-40% of females of reproductive age, assessment of serum CRP levels is not included in routine diagnostic for infertility evaluation so far (32).

Limitations of our study include the lack of a control blood test of serum ferritin levels and pregnancy rates or live birth rates after adequate iron intake. Furthermore, we were not able to obtain data about the ethnicity of our study cohort of infertile females, which might play a role regarding to iron intake as well. Despite, we assume that our sample size is adequate to examine substantial associations between serum ferritin levels and unexplained infertility. It should also be acknowledged that the age of the study participants differed between the two groups. However, when controlled for in the multivariate model, this fact did not appear to be correlated with the decreased ferritin levels (listed in **Table 3**). Furthermore, we could not provide information about the menstrual volume for participants of both groups which could have demonstrated hypermenorrhea-induced chronic blood loss resulting in iron deficiency.

In conclusion, our study showed an association of unexplained infertility with female serum ferritin levels <30µg/L. It needs to be emphasized that a single cutoff value for serum ferritin cannot be used to finally define iron deficiency, since not all individuals with a ferritin level <30µg/L will have other supportive evidence of iron deficiency. Moreover, evidence suggests some conditions respond to iron treatment despite ferritin levels above 30 (33, 34). Nonetheless, multiple indicators of low iron status were more prevalent in women with unexplained infertility. In addition, positive serum markers for thyroid autoimmunity were predictive markers for ferritin levels <30µg/L. Although the observations presented do not definitively establish a relationship between iron status and fertility, they provide a basis for future investigation. Such studies should clarify whether an assessment of iron status, including markers of inflammation, should be recommended in women with unexplained infertility. It will also be informative to investigate the possibility whether iron deficiency contributes to the effect of autoimmune thyroid disease on female fertility. Whether treatment for low iron status, when identified in this population, will improve fertility, remains speculative.

## Data availability statement

The raw data supporting the conclusions of this article will be made available by the authors, without undue reservation.

## Ethics statement

The studies involving human participants were reviewed and approved by Ethics Committee of the Medical University of Vienna, Vienna, Austria. The patients/participants provided their written informed consent to participate in this study.

## Author contributions

All authors contributed to conception and design of the study. IH, JO, and JE organized the database. IH, JO, and FH performed the statistical analysis. IH and JO wrote the first draft of the manuscript. All authors contributed to the article and approved the submitted version.
