# Supplementary material for: Iron status in women with infertility and controls: a case-control study
Source: Front Endocrinol (Lausanne). 2023 Jun 8;14:1173100. doi: 10.3389/fendo.2023.1173100 (PMC10285297; doi:10.3389/fendo.2023.1173100)
Supplement: Supplementary file 1 [file Table_1.docx]

**Supplementary Table**. Hematologic parameters in women with ferritin <30µg/L and ≥30µg/L.

|  | Ferritin <30µg/L  (n= 16) | Ferritin ≥30µg/L  (n= 56) | P |
| --- | --- | --- | --- |
| Erythrocyte count (T/L) * | 4.4 (4.1;4.8) | 4.5 (4.2;4.7) | 0.0773 |
| Hemoglobin (g/dL) * | 12.5 (11.9;13.5) | 13.4 (12.8;14.0) | 0.003 |
| Hematocrit (%) * | 37.9 (36.2;40.0) | 39.7 (38.2;40.7) | 0.500 |
| Mean corpuscular volume (fl) * | 88.2 (84.9;89.0) | 89.0 (86.2;91.6) | 0.009 |
| Mean corpuscular hemoglobin (pg) * | 29.5 (28.2;30.2) | 30.2 (29.1;31.1) | 0.005 |
| Mean corpuscular hemoglobin concentration (g/dL) * | 33.4 (33.0;33.9) | 34.0 (33.2;34.6) | 0.006 |
| Thrombocyte count (G/L) * | 270.5 (213.0;283.5) | 252.5 (220.8;285.0) | 0.471 |
| Leucocyte count (G/L) * | 5.8 (4.5;6.4) | 6.3 (5.0;7.3) | 0.146 |
| Iron (µg/dL) * | 60.0 (50.0;83.5) | 85.5 (59.5;102.5) | 0.091 |
| Transferrin (mg/dL) * | 301.0 (267.8;310.8) | 266.0 (242.0;283.5) | 0.006 |
| Transferrin saturation (%) * | 18.5 (12.3;26.7) | 20.6 (15.3;29.3) | 0.349 |
| C-reactive protein (mg/L) * | 0.60 (0.43;1.65) | 0.65 (0.33;1.50) | 0.703 |

Data are provided as median (IQR)
